# Supplementary material for: The causal role of circulating inflammatory markers in osteoporosis: a bidirectional Mendelian randomized study
Source: Front Immunol. 2024 Jul 18;15:1412298. doi: 10.3389/fimmu.2024.1412298 (PMC11291241; doi:10.3389/fimmu.2024.1412298)
Supplement: Supplementary file 1 [file DataSheet_1.zip › supplementary material/CIM-OSTEOPOROSIS /GCST90274795.csv_forest.pdf]

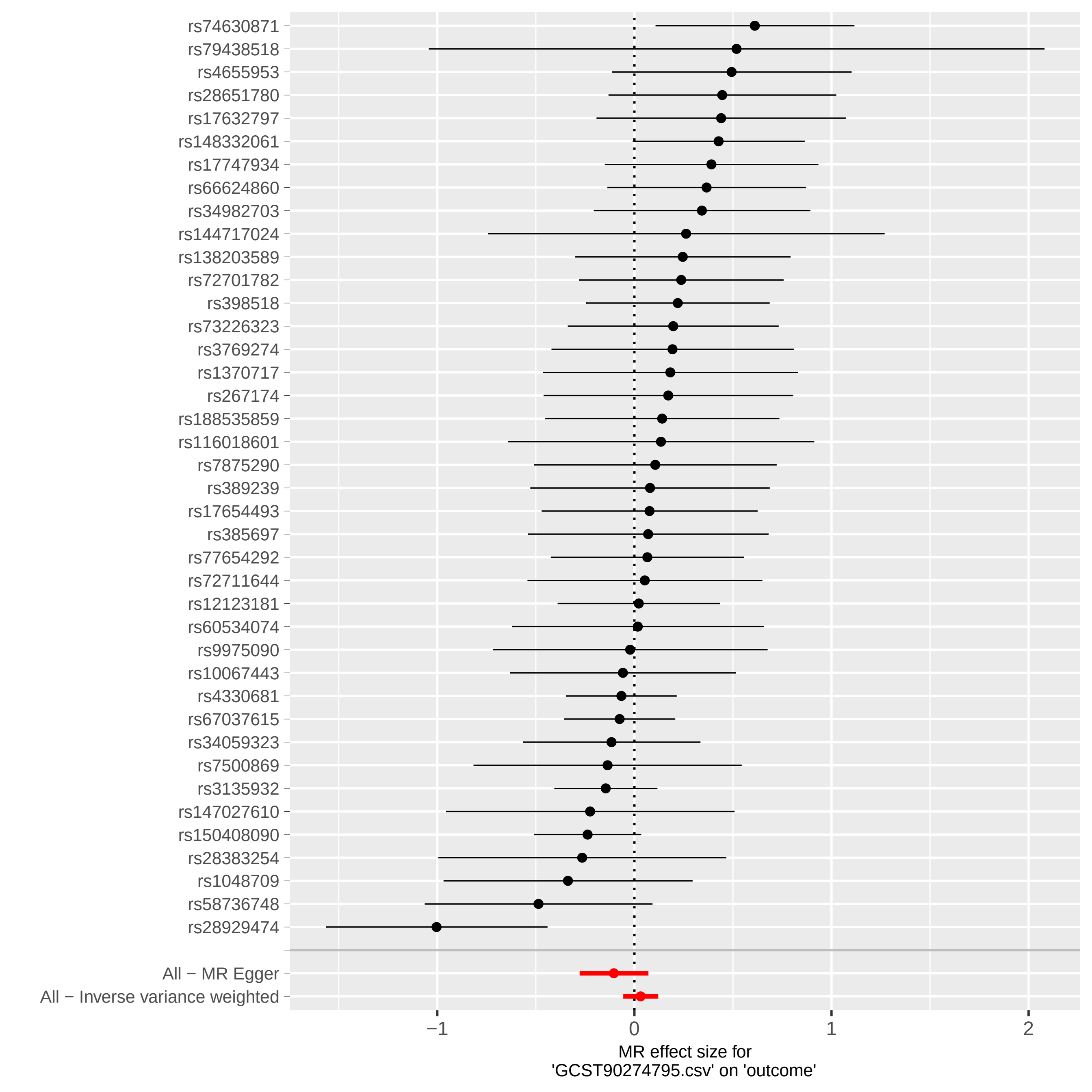
rs74630871
rs79438518
rs4655953
rs28651780
rs17632797
rs148332061
rs17747934
rs66624860
rs34982703
rs144717024
rs138203589
rs72701782
rs398518
rs73226323
rs3769274
rs1370717
rs267174
rs188535859
rs116018601
rs7875290
rs389239
rs17654493
rs385697
rs77654292
rs72711644
rs12123181
rs60534074
rs9975090
rs10067443
rs4330681
rs67037615
rs34059323
rs7500869
rs3135932
rs147027610
rs150408090
rs28383254
rs1048709
rs58736748
rs28929474
All − MR Egger
All − Inverse variance weighted
−1
0
1
2
MR effect size for
'GCST90274795.csv' on 'outcome'
